# Differentiation between temporary and real non-clearability of biotinylated IgG antibody by avidin in mice

**DOI:** 10.3389/fphar.2014.00172

**Published:** 2014-07-24

**Authors:** Shuping Dou, John Virostko, Mary Rusckowski, Dale L. Greiner, Alvin C. Powers, Guozheng Liu

**Affiliations:** ^1^Department of Radiology, University of Massachusetts Medical SchoolWorcester, MA, USA; ^2^Vanderbilt University Institute of Imaging Science, Vanderbilt UniversityNashville, TN, USA; ^3^Department of Molecular Medicine, University of Massachusetts Medical SchoolWorcester, MA, USA; ^4^Division of Diabetes, Endocrinology, and Metabolism, Department of Medicine, Vanderbilt UniversityNashville, TN, USA; ^5^Department of Molecular Physiology and Biophysics, Vanderbilt UniversityNashville, TN, USA; ^6^Veterans Affairs Tennessee Valley Healthcare SystemNashville, TN, USA

**Keywords:** pretargeting, antibody, clearing agent, avidin, *in vivo* accessibility, immunotargeting

## Abstract

Although an increasing number of antibody conjugates are being used in the clinic, there remain many unmet needs in antibody targeting. Normal tissue background is one of the key issues that limits the therapeutic efficacy and the detection sensitivity. Background reduction coupled with dose increase may provide the required target accumulation of the label or toxin at an acceptable normal tissue background. However, the knowledge about the *in vivo* interaction between antibody and a clearing agent is currently inadequate for designing a rational clearance regimen or system. The current investigation focuses on the clearability of antibody for background reduction, an important topic to antibody targeting in general. The investigation employs pretargeting as a research tool and avidin as a model clearing agent. By comparing the effects of natural clearance at a longer post-injection time and avidin clearance, we demonstrated that avidin clearance is much more effective. By directly attaching avidin to a biotinylated antibody prior to injection, we found that the biotinylated antibody in blood, once bound to the clearing agent, can be removed from the circulation immediately and completely, while the real non-clearable antibody without biotin stays. The study of multiple avidin injections confirmed that the presence of clearable biotinylated antibodies after an avidin injection is due to their temporary inaccessibility and subsequent return from tissue compartments. The collective clearance efficiency of 91% by three avidin injections indicates a continuous IV infusion would be recommended to remove all of the biotinylated IgG molecules. In conclusion, the use of antibody pretargeting as a tool in this study has improved understanding of the incomplete clearance by avidin and can aid in overcoming this obstacle.

## Introduction

For targeted immunotherapy and immunodiagnosis, the clearance of normal tissue background is an important measure complementary to the enhancement of target accumulation. Although targeted immunochemotherapy of hematological cancer has achieved great success (Senter and Sievers, 2012; Deng et al., 2013), the relative poorer accessibility of antibody to solid tumors remains a challenge. Reducing the normal tissue background may allow for increasing the dose of the “warhead” or the target toxicity and therefore may improve solid tumor treatment. The background reduction is also critical for imaging the islets of Langerhans (Liu et al., 2011, 2012). Because islets constitute only 1–2% of the pancreas mass and the current nuclear imaging technologies cannot differentiate islets from non-islet pancreatic tissues, reduction of the non-specific binding in the exocrine tissues is crucial to assure the pancreas signal reflects the beta cell accumulation.

Currently, there are two clearing mechanisms in the literature used for reducing the normal tissue background. One mechanism using a secondary-antibody takes advantage of the large size of the aggregate formed with the pretargeting antibody. The aggregate can be removed from the circulation by reticuloendothelial (RE) cells (Goodwin et al., 1994, 1988). The other mechanism employs a clearing agent bearing galactosyl groups. Such clearing agents can be avidin (Yao et al., 1995; Mirallie et al., 2005; Liu et al., 2010), galactosylated anti-antibodies against the pretargeting antibody (Sharkey et al., 1997), or galactosylated and biotinylated HSA (Axworthy et al., 2000). The complex formed between the antibody and clearing agent can be removed by an asialoglycoprotein receptor specific for the galactosyl groups (Ashwell and Morell, 1974; Ong et al., 1991). Both mechanisms traffic the circulating pretargeting molecules into liver. Most studies in the literature focus on the development of technologies that include a clearance step (Ashwell and Morell, 1974; Goodwin et al., 1988, 1994; Ong et al., 1991; Yao et al., 1995; Karacay et al., 1997; Sharkey et al., 1997; Axworthy et al., 2000; Wang et al., 2001; Mirallie et al., 2005; Liu et al., 2010). However, few efforts have been made to understand the *in vivo* interaction between the antibody and clearing agent (Kobayashi et al., 1995; Yao et al., 1995; Sharkey et al., 1997).

The clearance concept has been used for many years, but the current knowledge remains inadequate for readily designing a targeting system with a clearance step to achieve low blood background. The current investigation focuses on the clearability of biotinylated antibody using avidin as a clearing agent. It is known that avidin does not clear biotinylated antibody completely, but there is no quantitative study as to the exact cause. However, this topic is very important not only for developing a pretargeting technology with clearance, but also for any antibody-based drug for which the background is a concern.

In the current investigation, we employed a model pretargeting system to investigate the *in vivo* chemistry between avidin and biotinylated IgG antibody. The aim of this study is not to develop an improved pretargeting protocol but to understand the clearability of biotinylated antibody by avidin. As such, the pretargeting system is used as a research tool for understanding rather than solely for improved pretargeting technology. In this pretargeting system, the antibody CC49 is conjugated concomitantly with a biotin and a morpholino phosphorodiamidate oligomer (MORF). The biotin functions to bind to the clearing agent of avidin, whereas the MORF is for radiolabeling both *in vivo* and *in vitro*. In contrast to most clearance systems, the current design has avoided the binding competition between the clearing agent and the effector. In this investigation, normal mice are used to focus on the determinants of antibody clearance. Tumor accumulation will not be compromised by antibody clearance as previously concluded (Liu et al., 2010).

## Materials and methods

**Scheme 1:**
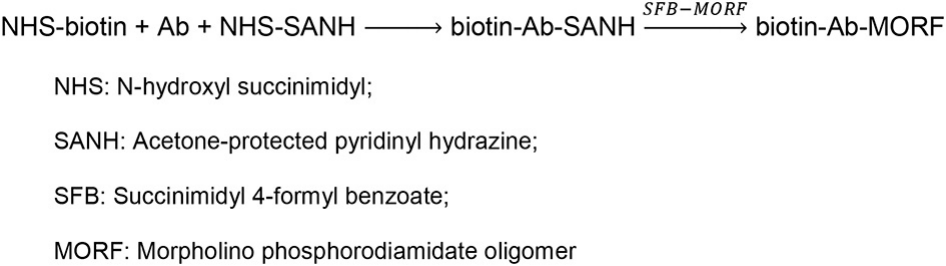
**The reactions of antibody conjugation with both biotin and MORF**.

The MORF and cMORF (complementary MORF) were custom-synthesized by Gene Tools, LLC (Philomath, OR). Their base sequences and amine derivatization were the same as previously reported (Liu et al., 2010). The EZ™ Biotin Quantitation Kit and avidin were from Pierce (Thermo Fisher Scientific, Rockford, IL). The model antiTAG-72 IgG antibody CC49 was prepared by Strategic Biosolutions (Ramona, CA) from its murine hybridoma cell line (a gift from Dr. Jeff Schlom, Center for Cancer Research, NCI, NIH). The biotin-CC49-MORF was synthesized following the reactions illustrated below (Scheme 1) and characterized as previously described (Liu et al., 2010).

The ^99 m^Tc-cMORF was prepared following a documented procedure (Liu et al., 2006). The ^99^Mo-^99 m^Tc generator was from Perkin Elmer Life Science Inc. (Boston, MA). All other chemicals were reagent grade and used without purification.

The concentrations of MORF and cMORF were measured by UV spectrophotometry using the molar absorbance values provided by the vendor. Size exclusion (SE) HPLC was used for the analyses of the MORF, cMORF and antibody. The HPLC system was equipped with a superpose-12 10/30 GL column (GE Healthcare Bio-Sciences AB, Uppsala, Sweden; optimal separation range: 1 × 10^3^ to 3 × 10^5^ Da), a UV in-line detector, and a radioactivity in-line detector. A 0.10 M pH 7.2 phosphate buffer was used as the eluant at a flow rate of 0.60 mL/min. Radioactivity recovery was routinely measured and was always greater than 90%.

We designed three animal studies in the current investigation. The animal use was approved by the IACUC of the University of Massachusetts Medical School. We first examined the natural background-reducing effect of time (prolonging pretargeting interval) and the use of avidin, to show the strength of clearing agent and to confirm the pharmacokinetic homogeneity of the antibody molecules after modification. We then studied the clearability of the biotin-CC49-MORF. The antibody was radiolabeled and bound to avidin prior to injection into mice, to mimic the avidin binding of antibody in blood. Based on the results from the first two studies, we designed a multiple avidin injection study to confirm that the biotinylated antibody that survived an avidin clearance was still clearable.

### Clearance efficiency by avidin with varying pretargeting interval

Because a longer wait (pretargeting interval prolongation) also reduces the non-specific background of antibody, we examined the effect of pretargeting interval prolongation along with avidin clearance. The statistical significance for the blood level changes by pretargeting interval prolongation and avidin clearance was analyzed by Two-Way analysis of variance (ANOVA) at the significant level of 0.05. A new quantity for the effect of avidin (clearance efficiency) is defined as the %ID/g reduction by avidin of blood radioactivity as compared to the %ID/g without avidin (100%—the blood level ratio with/without avidin injection). It can be calculated from the measured blood activity levels. The standard deviation of the clearance efficiency can be estimated by the uncertainty propagation formula:

$$SD=\sqrt{SD_{without}^{2}{\left(\frac{1}{Blood_{with}}\right)}^{2}+\;SD_{with}^{2}{\left(\frac{Blood_{without}}{Blood_{with}^{2}}\right)}^{2}}.$$

To label the antibody *in vivo*, the cMORF was injected in a many-fold molar excess as compared to the MORF on the antibody. The blood level of the cMORF was therefore proportional to that of the antibody and was a measure of the antibody blood level, but it is lower in value than the antibody level because only a small portion of the cMORF was consumed and the rest of the cMORFs were excreted rapidly. The relationship is: %ID/g_antibody_ = %ID/g_cMORF_/(molar dose ratio of cMORF/antibody × MORF number per antibody).

**Scheme 2:**
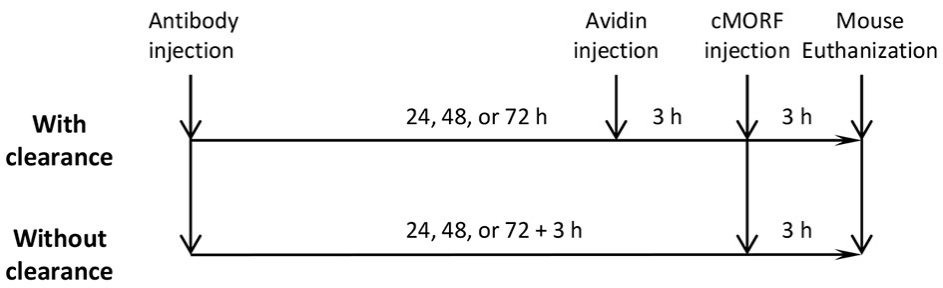
**The study design examining the clearance effect of longer wait and avidin injection**.

As shown in Scheme 2, in a study set of 3 groups of mice (*N* = 4), each of the 12 mice received via tail vein 30 μg of biotin-CC49-MORF (0.67 MORFs and 3.41 biotins per antibody); 1, 2, and 3 days later, an avidin dose of 34 μg was administrated intravenously to the 3 groups of 4 mice, followed 3 h later by the injection of 1.2 μg (80 μCi) of ^99 m^Tc-cMORF. The mice were euthanized for biodistribution at 3 h subsequent to the radioactivity injection. In a control study set, an otherwise identical procedure was applied except for the removal of the avidin injection. The 1.2 μg of cMORF was an optimized mass dose that was sufficient to saturate the MORF-antibody and the 34 μg of avidin was previously determined to be more than sufficient to saturate the biotin-modified MORF-antibodies in the circulation (Liu et al., 2010). The decrease of blood radioactivity level of ^99 m^Tc-cMORF was used as the measure of background improvement as it was proportional to the decrease of the MORF-antibody level.

### Avidin clearability of the biotin-antibody-morf

As will be shown, although avidin clearance was much more effective as compared to the prolonged pretargeting interval, the clearance effect was still considerably low (50–60%). Thus, we sought to determine whether a biotinylated antibody could be cleared completely once bound to avidin in blood. We designed a study to inject biotinylated antibody both radiolabeled and bound to avidin prior to the IV injection, to mimic the antibody in blood immediately after binding to avidin. Thus, a biotin-CC49-MORF antibody (0.63 MORFs and 1.68 biotins per antibody) was radiolabeled by mixing with ^99 m^Tc-cMORF (at a molar ratio of MORF/cMORF = 3/1). The mixture was purified over a G-100 size-exclusion column. The radiolabeled biotin-CC49-MORF was then allowed to combine with excess avidin (molar ratios of avidin/biotin = 5). The pharmacokinetics of the both radiolabeled and avidin-bound antibody was then measured. Mice in 4 groups (*N* = 4, each group) each received an IV injection of the mixture containing 12.5 μg (9 μ Ci) of ^99 m^Tc-labeled biotin-CC49-MORF and 45 μg of avidin. At 2 min, 30 min, 1.5 h, and 2.5 h, the mice were euthanized for measuring the biodistribution. In a control set also consisting of 4 groups (*N* = 4, each group), an identical procedure was performed except avidin was not added to the injectate.

### Multiple injections of avidin

**Scheme 3:**
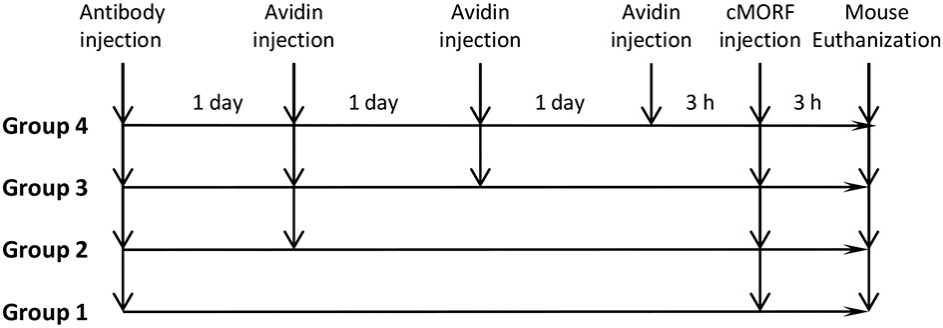
**The study design for the pretargeting regimens with multiple injections of avidin**.

As will be shown, the clearance efficiency in the first study was less than the clearable portion measured in the second study. We thus sought to determine whether the non-cleared portion is temporary non-clearable due to the sequestration of the antibody in the extravascular space. As shown in Scheme 3, each mouse of 4 groups (*N* = 4) was injected with 30 μg of biotin-CC49-MORF (0.63 MORFs and 1.68 biotins per antibody). One day after antibody injection, mice in groups 2, 3, and 4 were injected with 34 μg of avidin. Two days later, the mice in groups 3 and 4 received another avidin injection. Three days later, mice in group 4 were given the third avidin injection. At 3 h following the last avidin injection of group 4, all mice in groups 1–4 received the same dose of radiolabeled cMORF (1.1 μg, 40 μCi) and all mice were euthanized for biodistribution at 3 h after the cMORF injection. The clearance effect for each avidin injection was estimated by comparing the group with that avidin injection as the last avidin injection to another group without it (group 1 vs. 2 for the first avidin injection, group 2 vs. 3 for the second, and group 3 vs. 4 for the third).

## Results

### Clearance efficiency by avidin with varying pretargeting interval

**Figure 1:**
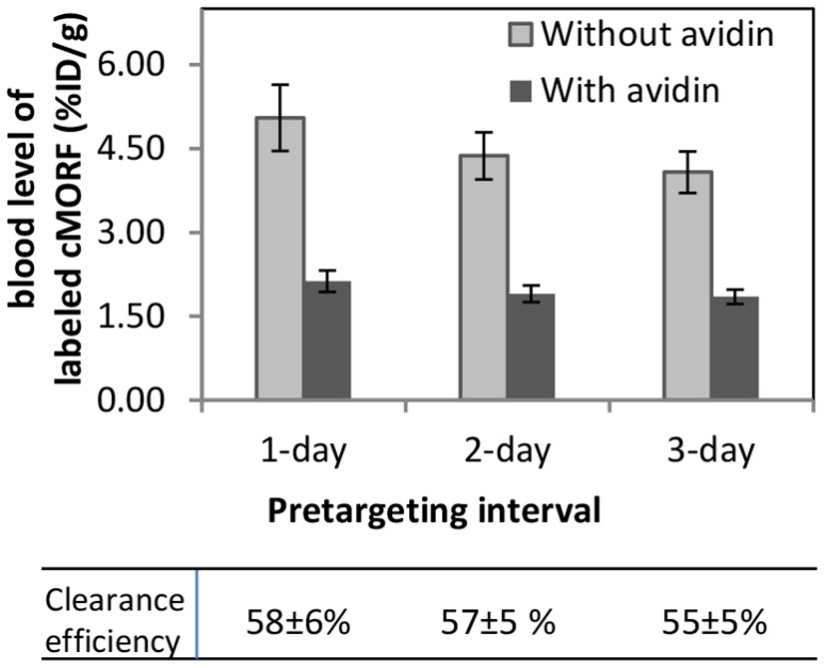
**Clearance efficiency at different pretargeting intervals in a pretargeting procedure using biotin-CC49-MORF, avidin, and ^99 m^Tc-cMORF**. The cMORF level is proportional to and used as a measure of the antibody level. The clearance efficiency is defined as the ratio of blood level with/without avidin.

Both prolonging the pretargeting interval and the use of avidin reduced the antibody levels in blood as shown in Figure 1 and normal tissues (not presented), but the use of avidin is more effective. Two-Way ANOVA indicates that the cMORF blood level is significantly decreased both by pretargeting interval prolongation (*p* = 0.007) and avidin administration (*p* < 0.00005). With regard to avidin administration, the result of ANOVA is in agreement with the visual judgment based on the large difference relative to the SD at each interval. The clearance efficiency using avidin as a clearing agent is calculated to be 55–58%, with >40% antibody remaining in the circulation. As will be shown, this incomplete clearance is neither due to ineffective binding of avidin nor to the dissociation of the avidin/biotin complex but mainly results from the rapid clearance of avidin from blood to liver.

The cleared and the remaining labeled molecules in the circulation seem to share the same pharmacokinetics. The varying pretargeting interval did not change the clearance efficiency (Figure 1). No significant difference is evident among the clearance efficiencies at the 1–3 day pretargeting intervals.

### Avidin clearability of the biotin-antibody-morf

**Figure 2:**
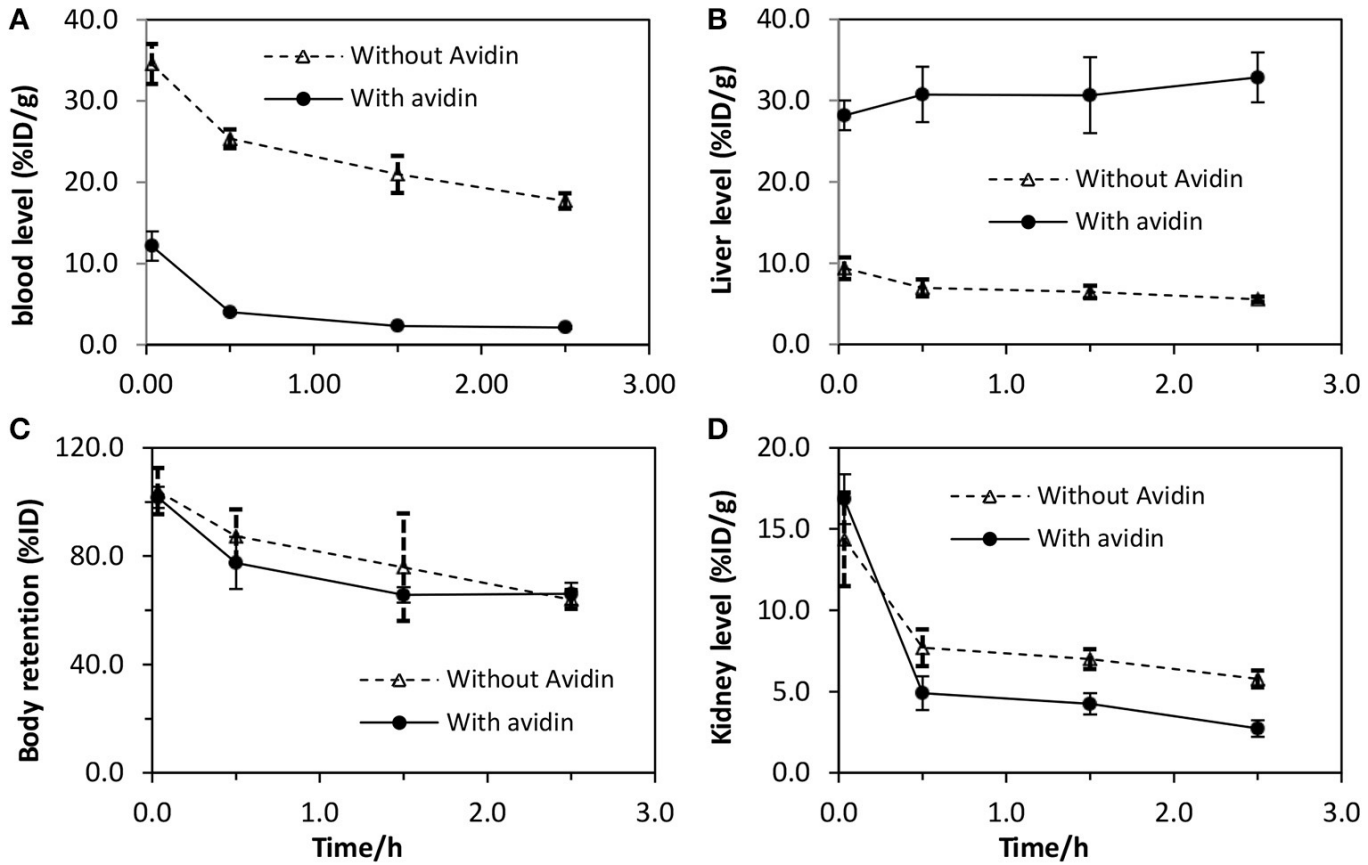
**Pharmacokinetics of both avidin-bound and radiolabeled biotin-CC49-MORF vs. radiolabeled biotin-CC49-MORF (no avidin)**. Shown are pharmacokinetics for **(A)** Blood, **(B)** Liver, **(C)** whole body retention, and **(D)** kidney. It mimics how a biotinylated antibody molecule behaves once bound to avidin in blood.

As shown in Figure 2A, the blood concentration of the radiolabeled biotin-CC49-MORF not treated with avidin is much higher than that for the avidin-treated. The percentages cleared from circulation (1-the ratio of blood levels with/without avidin) are 65 ± 3%, 84 ± 1%, 89 ± 1%, and 88 ± 1% for 2, 30 min, 1.5, and 2 h, respectively. At 0.5 h, the clearance of the avidin-treated antibody is almost complete, although additional slight improvement is observed until 1.5 h. Thus, about 88% of the avidin-treated biotin-CC49-MORF is clearable. The approximate 12% of avidin that is non-clearable (that cannot be cleared by avidin) is likely due to the lack of a biotin group.

The high liver accumulation in Figure 2B confirms the previous report that avidin clears the antibody into this organ (Ashwell and Morell, 1974; Ong et al., 1991). Subsequent clearance out of this organ is slow, as shown in Figure 2C by the almost identical whole body retention. The ~30% loss of the radioactivity in the whole body is likely due to the lower affinity of some sequence impurities and therefore the dissociation of the MORF/cMORF complex. Figure 2D demonstrates higher kidney accumulations after 30 min for the non-avidin-treated antibody as compared to the avidin-treated, likely due to the higher blood concentrations of the antibody shown in Figure 2A.

### Multiple injections of avidin

**Figure 3:**
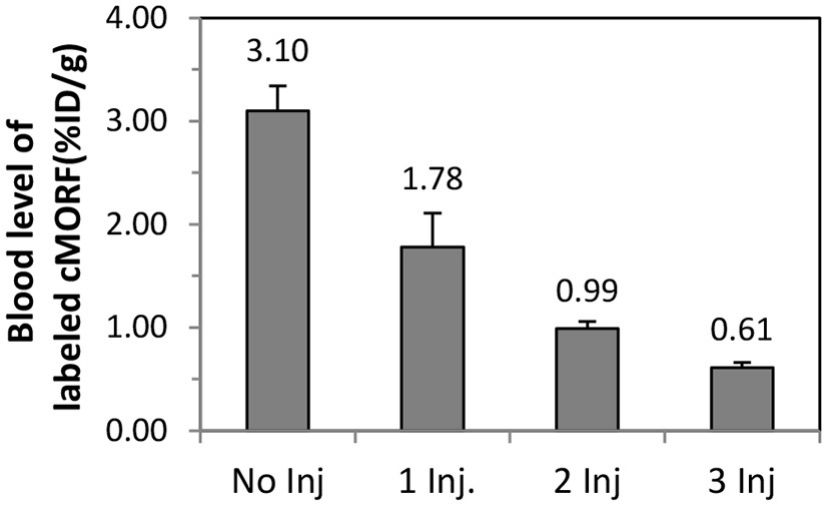
**The blood radioactivity levels after the injection of the biotin-Ab-MORF, different numbers of injections of avidin, and the injection of the radiolabeled cMORF**. The time of euthanization was at 4 days after initial injection of the pretargeting antibody and 3 h after injection of radiolabeled cMORF (see Materials and Methods).

As shown in Figure 3, additional injections of avidin subsequent to the first further reduce the blood antibody concentration. This indicates that the majority of antibody molecules that survived the first avidin injection are clearable. The collective clearance efficiency following 3 avidin injections is 80 ± 2% (0.61/3.10^*^100%). The separate clearance efficiency following each injection is 43 ± 12% (1 − 1.78/3.10), 44 ± 11% (1 − 0.99/1.78), and 38 ± 7% (1 − 0.61/0.99), respectively. The real clearance efficiency may actually be higher because of the non-clearable portion. A calculation based on a constant clearance efficiency of 51% (higher than 38–44%), and the non-clearable portion of 12% provides the blood concentrations of 3.10, 1.71, 1.03, 0.69%ID/g. These numbers are in a good agreement to the measured 3.10 ± 0.24, 1.78 ± 0.33, 0.99 ± 0.07, 0.61 ± 0.05%ID/g.

## Discussion

In the current investigation, we have demonstrated that biotinylated antibody can be cleared from the circulation completely and rapidly if bound to avidin. To the best of our knowledge, until this investigation it remained a hypothesis, although the incomplete clearance of antibody by avidin was known. Yao et al. hypothesized that one of the possible reasons underlying the incomplete clearance might be a too low biotinylation of the antibody (Yao et al., 1995), because avidin would not bind an antibody without biotin. In the current investigation, this possibility was confirmed to be limited to only ~12% for the MORF-Ab carrying an average of 1.68 biotins per molecule. Another possible explanation Yao et al. proposed was the inaccessibility of the antibody that diffused into the interstitial fluid (extravascular space). As some clearing agents reached a clearance effect of >90% (Sharkey et al., 1997; Axworthy et al., 2000), an auxiliary explanation may be the rapid clearance of avidin relative to the diffusion into the extravascular space (Mirallie et al., 2005). It has been reported that most labeled avidin is eliminated from blood within 2 min in rabbits (Rosebrough, 1993). When the avidin is essentially cleared from circulation, the extravascular antibodies would return. A rebound of the radiolabeled antibody concentration in the blood has been previously observed (Kobayashi et al., 1995).

The hypothesis that temporary inaccessibility of the antibody in the extravascular space, coupled with the rapid clearance of avidin from the circulation, also explains the avidin dose-clearance curves (Rosebrough, 1993; Liu et al., 2010). An initial narrow dose range with efficient improvement in antibody clearance may correspond to the range of saturating the biotinylated antibody within the blood pool. The subsequent less effective larger range may reflect the difficulty for the avidin to diffuse across the capillary wall into the extravascular space due to its rapid clearance from the blood.

We have employed a pretargeting system to measure the concentration of the antibody in the current investigation as well as previous studies (Liu et al., 2010). The conventional direct-labeling approach (to compare labeled antibody level in normal tissues with and without an injection of clearing agent) would underestimate the clearance efficiency, because radiolabels, particularly radiometals, may have dissociated from antibody and localized within the cells. In this study, we have defined the clearance efficiency. It can also be calculated based on the measurement by pretargeting.

The immediate utility of the findings in this investigation is that it paves the way for using avidin as the clearing agent to develop an improved pretargeting technology. The tri-functional antibody allows for independent binding to the target, to the clearing agent, and to the labeled effector. In some other pretargeting technologies with galactosylated clearing agent, the effector and the clearing agent compete for the same binding site. Although the clearance efficiency by a single injection of avidin is lower than that provided by a single injection of secondary antibody (50–60% vs. >90%), it would not affect the endogenous IgG antibodies in animal and human (Sinitsyn et al., 1989). As the lower clearance efficiency (50–60%) stems from the too rapid clearance of avidin, a continuous infusion within 1 or 2 h would achieve a clearance effect suitable for clinical applications.

More importantly, the topic of antibody-trafficking among different compartments and its clearance from circulation are of interest for immunotherapy or targeted chemotherapy ingeneral. The clearance approach may apply similarly to the antibody-drug conjugates for tumor targeting. Clearance of the circulating antibody would greatly reduce the toxicity to normal tissues and therefore may permit higher dose for increased toxicity to the tumor.

## Conclusion

Understanding the *in vivo* interaction between antibodies and their clearing agent should be useful for antibody-based drug discovery. We demonstrated the rapid clearance from blood of every avidin-bound antibody molecule, differentiated the temporary non-clearable and the real non-clearable antibody molecules, and validated the previous hypotheses in the literature. We also quantitated the clearance processes by defining clearance efficiency and using pretargeting as a research tool. Based on the multiple injection study, we suggest a continuous administration (infusion) of the clearing agent as a solution for the temporary non-clearable antibodies.

### Conflict of interest statement

The authors declare that the research was conducted in the absence of any commercial or financial relationships that could be construed as a potential conflict of interest.
